# The Adipokines in Cancer Cachexia

**DOI:** 10.3390/ijms21144860

**Published:** 2020-07-09

**Authors:** Michele Mannelli, Tania Gamberi, Francesca Magherini, Tania Fiaschi

**Affiliations:** Department of Biomedical, Experimental and Clinical Sciences, University of Florence, Viale Morgagni 50, 50134 Florence, Italy; michele.mannelli@studenti.unisi.it (M.M.); tania.gamberi@unifi.it (T.G.); francesca.magherini@unifi.it (F.M.)

**Keywords:** adipose tissue, cancer cachexia, adipokines

## Abstract

Cachexia is a devastating pathology induced by several kinds of diseases, including cancer. The hallmark of cancer cachexia is an extended weight loss mainly due to skeletal muscle wasting and fat storage depletion from adipose tissue. The latter exerts key functions for the health of the whole organism, also through the secretion of several adipokines. These hormones induce a plethora of effects in target tissues, ranging from metabolic to differentiating ones. Conversely, the decrease of the circulating level of several adipokines positively correlates with insulin resistance, metabolic syndrome, diabetes, and cardiovascular disease. A lot of findings suggest that cancer cachexia is associated with changed secretion of adipokines by adipose tissue. In agreement, cachectic patients show often altered circulating levels of adipokines. This review reported the findings of adipokines (leptin, adiponectin, resistin, apelin, and visfatin) in cancer cachexia, highlighting that to study in-depth the involvement of these hormones in this pathology could lead to the development of new therapeutic strategies.

## 1. Adipose Tissue

Adipose tissue has long been considered as an inert organ, primarily storing energy in the form of lipids to be used as an energy source. Indeed, adipose tissue efficiently stores surplus energy in the form of neutral triglycerides (TGs) and controls lipid mobilization, thus modulating whole-body energy balance [1]. However, in the last decades, adipose tissue has emerged as a dynamic and heterogeneous endocrine organ with inflammation-modulating activity, responsible for the synthesis and secretion in the bloodstream of several active molecules, playing a crucial role in the regulation of whole-body metabolic homeostasis [2]. Adipose tissue actively regulates crucial biological processes by modulating feeding, total energy expenditure, hematopoiesis, and overall immune function [2,3]. Consequently, dysfunctions in this compartment lead to several metabolic and systemic diseases. According to anatomic location, adipose tissue depots are divided into subcutaneous and visceral subtypes. In humans, subcutaneous depots, contained primarily in abdominal, gluteal, and femoral regions, represent about 80% of total body adipose tissue. Visceral adipose tissue depots (surrounding organs and divided in mesenteric, gonadal, epicardial, retroperitoneal, omental, and perirenal fat depots) represent from 5% to 20% of total body fat in normal-weight individuals. Small adipose tissue depots, such as intramuscular and bone marrow adipose tissue, exert protection and support functions [4].

White adipose tissue (WAT) and brown adipose tissue (BAT) are distinguished on the basis of adipocyte origin, morphology, mitochondria amount, and thermogenic gene expression. WAT constitutes the largest component of adipose tissue, containing large and spherical adipocytes arising from resident cells of mesenchymal origin (Myf5−), endowed of single lipid droplet occupying most of the cell volume, few mitochondria, and a low oxidative rate [5,6]. WAT is specialized in the storage and release of lipids. Indeed, WAT stores TGs that are degraded through lipolysis activation, thus fueling energy-demanding tissues with glycerol and fatty acids in metabolic stress conditions [7]. In addition to its lipid-storing and mobilization capacity, WAT has been described as a crucial endocrine organ able to modulate a wide variety of biological and physiological processes [8]. In agreement, dysfunctions in WAT compartments lead to severe metabolic disorders and syndromes, including obesity, lipodystrophy, and cachexia [9,10].

Brown adipose tissue is composed of adipocytes containing multilocular lipid droplets, high content of mitochondria, elevated lipid oxidation activity, and widespread vascularization [10]. Although, for a long time, functional BAT was suggested to be present only in young individuals and rapidly undergoing to involution with age, small BAT depots have been found in adults, located close to aorta and kidney and within the supraclavicular region of the neck [5]. In addition to the origin from skeletal muscle-like lineage Myf5+ precursors, recent data demonstrate that BAT originates from Myf5− precursors, thus highlighting the complexity of adipocyte origin [11]. The functional hallmark of BAT is the energy dissipation. This is largely due to a proton leakage pathway mediated by uncoupling protein 1 (UCP-1). Uncoupling protein 1, strongly overexpressed in brown adipocyte mitochondria, uncouples oxidative phosphorylation from ATP synthesis in the inner mitochondrial membrane, thus dissipating energy in heat [12].

More recently, a new type of adipocyte defined “brite” (brown-in-white), also referred to as “beige”, has been identified in both subcutaneous and visceral WAT. Although arising from white adipocyte cell lineage (Myf5− precursors), brite adipocytes express UCP-1 and have similar functions to brown adipocytes [13]. Currently, many authors support the model of trans-differentiation, suggesting that the brite thermogenic adipocytes could develop from pre-existing white adipocytes in response to several endocrine and paracrine stimuli able to trigger the white-to-brite transition, by a process referred to as “browning” [14,15]. Cold stress and β3-adrenoreceptor agonists have been addressed as responsible for the shift from white to brite adipocytes in WAT [16]. However, de novo generation of brite adipocyte in WAT from Myf5− precursors has been highlighted. Indeed, adipocyte progenitors in WAT can be committed into either white or brite adipocyte precursors, depending on extracellular stimuli. For example, interleukin 4 receptor α (IL4Rα) signaling guides the commitment of adipocyte progenitors to brite adipocyte precursors [17,18]. Considering their thermogenic function, as well as plasticity, brite adipocytes, and particularly white-to-brite transition, have been shown to be involved in the disrupted energy balance observed in metabolic syndromes as cancer-associated cachexia.

### 1.1. Adipogenesis

Through adipogenesis, committed immature adipose cells (pre-adipocytes) differentiate into mature adipocytes. Both morphological and functional changes occurring during adipogenesis are essentially due to transcription factor cascade activation that coordinates the expression of genes responsible for adipocyte function [19]. Besides, the extracellular matrix (ECM) plays a crucial role in adipocyte differentiation. Indeed, extracellular stimuli, and particularly ECM remodeling, drives the transcriptional factor network activity responsible for adipocyte differentiation [20]. This tight regulated process leads immature pre-adipocytes to a final mature phenotype characterized by cell shape rearrangement and enhanced lipid accumulation [21]. Adipose tissue expansion, i.e., pre-adipocytes differentiation into mature adipocytes, is allowed throughout life, thus enabling increased storage of surplus energy when needed. Furthermore, adipocytes can adapt their size to satisfy the increased/decreased storage requirements, modulating their shape and number in response to energy balance [22].

**Figure 1:**
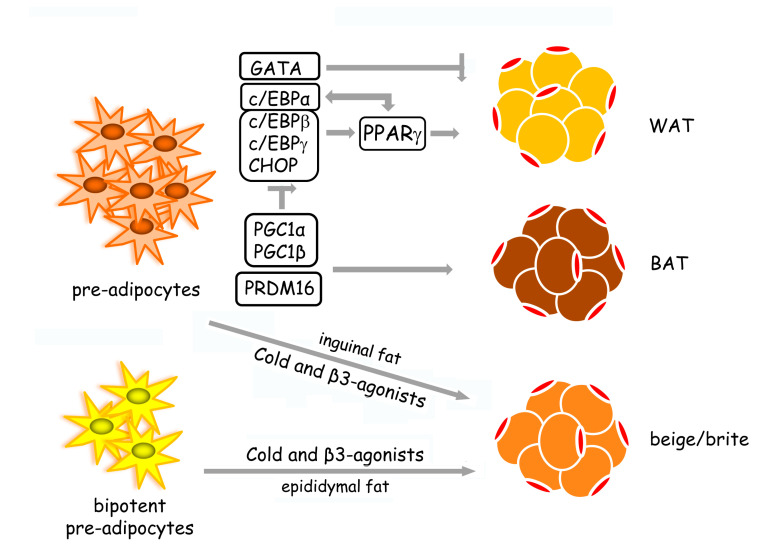
The transcriptional network that guides adipogenesis. White adipogenesis (WAT) is controlled through PPARγ-dependent mechanisms. The pro-adipogenic factors—c/EBPα, c/EBPβ, c/EBPγ, and CHOP—activate adipogenesis, while GATA transcription factors act as anti-adipogenic factors. PPARγ up-regulates c/EBPα that, in turn, promotes PPARγ expression. Brown adipocyte differentiation (BAT) is induced by the transcriptional co-regulator PRDM16. In addition, PPARγ co-activators—PGC1α and PGC1β—promote brown adipocyte formation by inhibiting the c/EBP network. In inguinal fat, cold stress and β-adrenergic stimulation directly induce the differentiation of pre-adipocytes in beige/brite adipocytes, while in epididymal fat, the same stimuli promote the formation of the beige/brite adipocytes from bipotent pre-adipocytes able to differentiate also in WAT under excess caloric condition [16].

Transcriptional pathways activated during adipogenesis have been well studied (Figure 1). The nuclear receptor peroxisome proliferator-activated receptor γ (PPARγ) acts as an adipogenesis master regulator. Pro-adipogenic factors, such as c/EBPs (homologous to CCAAT/enhancer-binding proteins), Krüppel-like factors (KLFs), and anti-adipogenic factors as GATA transcription factors, regulate adipogenesis via PPARγ-dependent mechanisms [19]. In particular, c/EBPs transcription factors (including α, β, γ, δ, and c/EBP homologous protein (CHOP)) coordinate and regulate adipogenesis through PPARγ. PPARγ up-regulates c/EBPα, which, in turn, promotes PPARγ expression, as well as other adipogenic genes [23]. Furthermore, PPARγ is also required for the maintenance of adipocyte-differentiated phenotype [19]. The zinc-finger transcriptional co-regulator PR domain containing 16 (PRDM16) has emerged as a key driver of brown adipocyte differentiation. Indeed, PRDM16 suppresses white adipocyte-specific genes by forming complexes with C-terminal binding proteins 1 and 2 (CTBP1 and CTBP2) [24]. PPARγ co-activators—peroxisome proliferator-activated receptor gamma coactivator (PGC)-1α and PGC1β—participate in brown adipocyte differentiation by impairing c/EBPs network and promoting the expression of brown-specific genes [25].

### 1.2. Lipid Homeostasis in Adipose Tissue

Adipose tissue stores triglycerides (TGs) produced through lipogenesis and releases fatty acids by activation of lipolysis. Hence, WAT lipid homeostasis, which is a key regulator of whole-body energy metabolism, is determined by the tight regulation between lipogenesis and lipolysis. Feeding stimulates lipogenesis and TG accumulation in adipose tissue depots, whereas fasting promotes lipolysis, thus leading to TG reserve breakdown and free fatty acids release in the bloodstream.

Lipogenesis is de novo fatty acid synthesis starting from acetyl-coenzyme A (Acetyl-CoA), and the esterification with a glycerol molecule to obtain TGs, represents an exceptionally efficient fuel storage molecule. Lipid storage is under tight hormonal control, and insulin acts as the master regulator of the process. In the feeding state, a high circulating level of glucose promotes lipogenesis by stimulating the release of insulin from the pancreas. In this condition, insulin increases adipocyte glucose uptake from the bloodstream by the insulin-dependent glucose transport 4 (GLUT 4), activates glycolytic and lipogenic enzymes, and stimulates the expression of the lipogenic gene sterol regulatory element-binding protein 1 (SREBP1) that regulates the expression of genes required for lipid synthesis [26]. Glucose provides new Acetyl-CoA as a substrate for de novo lipogenesis and induces the expression of Acetyl-CoA carboxylase (ACC) that represents the rate-limiting enzyme of the process. Fatty acids are finally esterified with a molecule of glycerol-3-phosphate to form lipid droplets of TGs. In normal conditions, fatty acids used for TG biosynthesis within adipocytes mainly derive from the bloodstream, while glycerol derives from the circulating TGs within chylomicrons and very-low-density lipoproteins. Lipoprotein lipase (LPL), secreted by adipocytes and translocated in the lumen of WAT capillaries, plays a crucial role in facilitating the release of fatty acids from circulating TGs into adipocytes [27]. Insulin induces the expression of the fatty acid transport gene and triggers LPL activation, thus enhancing fatty acid uptake and esterification [27,28].

Conversely, TGs are hydrolyzed to glycerol and free fatty acids through lipolysis. Lipolysis supplies glycerol for hepatic gluconeogenesis and free fatty acids for oxidation in energy-demanding tissues. Indeed, this process is activated by fasting or other metabolic stress conditions (i.e., strenuous exercise) when is necessary to satisfy the energy demands of tissues [28]. TG breakdown is primarily guided by adipocyte triglyceride lipase (ATGL) and hormone-sensitive lipase (HSL). These enzymes are responsible for the conversion of TGs to diglycerides and then to monoglycerides. Then, monoacylglycerol lipase (MAGL) triggers the hydrolysis of the third fatty acid and glycerol release. As lipogenesis, lipolysis is also tightly regulated. During fasting, decreased circulating levels of insulin lead to lipogenesis suppression and the activation of the lipolytic pathway. This is mainly driven by the elevated amount of circulating hormones like glucagon and catecholamine (highly released by the sympathetic nervous system during fasting) that are responsible for protein kinase A (PKA) activation [29]. PKA phosphorylates surface lipid droplet-associated proteins, such as perilipins, thus enhancing HSL activity [30]. Particularly, HSL, inhibited by insulin and stimulated by epinephrine and glucagon, has been highlighted as a key regulator of lipolysis [28].

## 2. Adipose Tissue in Cancer Cachexia

Cancer cachexia is a devastating and systemic syndrome characterized by the progressive bodyweight loss mainly due to skeletal muscle wasting and atrophy occurring in response to cancer growth [31]. Cancer-associated cachexia occurs in up to 50–80% of end-stage cancer patients, accounting for more than 20% of all cancer-related deaths [32]. Among organs, skeletal muscle and adipose tissue represent the primary targets during cancer cachexia progression, characterized by metabolic alterations related to carbohydrates, lipids, and proteins [33]. Interestingly, some findings suggest a tight adipose tissue-skeletal muscle crosstalk during cancer cachexia [34].

Although skeletal muscle wasting due to increased protein breakdown is known as the major hallmark of cancer cachexia, the depletion and remodeling of adipose tissue is a crucial effect in cachectic patients. Recently, adipose tissue wasting has been shown to occur before the appearance of other classical cachexia markers (i.e., fat loss is a more rapid event in comparison to lean mass depletion) [35,36]. In addition, adipose tissue of cachectic cancer patients has been highlighted as a potential source of pro-inflammatory cytokine production during cancer cachexia progression [37].

### 2.1. Morphological Remodeling of Adipose Tissue in Cancer Cachexia

**Figure 2:**
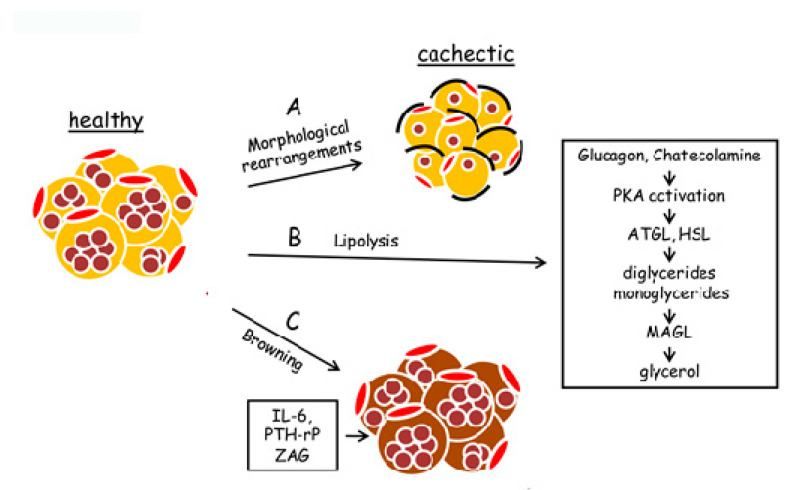
Modifications, occurring in adipose tissue in cancer cachexia. Cancer cachexia provokes both phenotypic and metabolic modifications in adipose tissue. (**A**) Healthy adipocytes undergo morphological rearrangements, ranging from a decrease of cell volume to fibrosis formation. (**B**) Cachectic adipocytes are characterized by enhanced lipolysis, leading to a considerable intracellular triglyceride breakdown. Lipolysis is activated by hormonal signals (as glucagon and catecholamine) that activate protein kinase A (PKA). PKA enhances adipocyte triglyceride lipase (ATGL) and hormone-sensitive lipase (HSL) activity. These enzymes induce the cleavage of two fatty acids from glycerol, forming diglycerides and monoglycerides. Monoacylglycerol lipase (MAGL) induces the hydrolysis in monoglycerides of the third fatty acid, thus forming free glycerol. (**C**) “Browning” of adipose tissue is induced by the host or tumor-derived pro-inflammatory factors as interleukin-6 (IL-6), tumor-derived parathyroid hormone-related protein (PTH-rP), and tumor secretory factor zinc 2-glycoprotein (ZAG). Brown adipocyte is characterized by a high amount of mitochondria and an enhanced level of uncoupling protein 1 (UCP-1) that dissipates energy in heat, uncoupling oxidative phosphorylation from ATP synthesis.

Adipose tissue remodeling is associated with several chronic diseases, including cancer cachexia. These changes comprise morphological and structural modifications characterized by adipocyte atrophy due to impairment of several processes as increased lipid mobilization, enhanced triglyceride lipolysis [35], reduced lipogenesis [38], impaired adipogenesis, [39], extracellular matrix (ECM) rearrangement (generally resulting in fibrosis) [40], enhanced inflammation [41], and adipose tissue “browning” [42]. The main morphological and biochemical modifications induced by cancer cachexia in adipose tissue are shown in Figure 2.

#### 2.1.1. Adipose Tissue Atrophy

Decrease of the adipocyte area and the perimeter alteration are the main morphological changes observed in both animal models and cachectic patients [37]. Cachectic mice bearing colon adenocarcinoma MAC16 clearly showed that adipocyte remodeling went beyond adipocyte size reduction and was linked to other morphological rearrangements. Adipose tissue of these mice showed shrunken adipocytes of various sizes contained several smaller lipid droplets surrounded by altered mitochondria, irregular cell outlines, and dilated interstitial space enriched of capillary vessels. Moreover, the formation of fibrosis and infiltration of inflammatory cells are also involved in architectural modifications in cachectic adipose tissue [40]. Morphological alterations are dependent on the type of adipose tissue involved (i.e., visceral or subcutaneous). Cachectic patients with gastrointestinal cancer manifest a relevant visceral adipose tissue depletion when compared to healthy individuals. Results obtained using Walker 256 breast carcinoma-bearing mice as cachexia experimental animal model showed that inflammation, ECM rearrangement, and metabolic alterations linked to unbalance of lipid turnover occurred differently in subcutaneous and visceral adipose tissue depots [43].

Although skeletal muscle atrophy represents the critical component of the body mass loss observed in cancer cachexia, the breakdown of adipose tissue through the promotion of lipolysis has been suggested as an important step in the onset of cancer cachexia. That lipolysis plays a key role in the onset of cachexia is suggested by observing that cachectic patients show high circulating levels of those factors promoting lipid mobilization, as zinc 2-glycoprotein (ZAG) [44]. ZAG, sharing high sequence homology with lipid mobilization factor (LMF), is secreted by tumor under cachectic conditions in order to stimulate triglyceride hydrolysis. ZAG strongly stimulates lipolysis by the activation of TAG lipase and up-regulates UCPs, thus promoting fatty acid oxidation in adipocytes [45]. Hence, ZAG is greatly involved in the formation of high circulating levels of free fatty acids and increases energy expenditure observed in cachectic cancer patients [36]. In agreement, both animal models and cachectic patients show ZAG up-regulation [46]. Interestingly, the high circulating levels of free fatty acids have been correlated with the worsening of muscle protein breakdown observed in cancer cachexia.

Overall, adipose tissue atrophy due to increased lipid mobilization can be considered a crucial point in the onset and development of cancer cachexia, strongly contributing to the negative energy balance and promoting skeletal muscle wasting observed in cachectic patients [47,48].

#### 2.1.2. Adipogenesis Impairment

Several metabolic and inflammatory pathways are involved in adipose tissue remodeling during cancer cachexia. Whether cachexia-mediated effects observed in adipose tissue are due to tumor-derived or host- tumor-induced products is not completely clear, so far. In this regard, an in vitro study highlights that the co-culture of Lewis lung carcinoma cells impairs in vitro adipogenesis of 3T3-L1 cells by promoting relevant lipid droplet volume reduction. Increased pro-inflammatory cytokine secretion by both tumor cells and adipocytes, as well as down-regulation of adipogenic genes, has been observed, thus suggesting that tumor cells could be able to impair adipocyte maturation by triggering an inflammatory response mediated by both tumor and adipocytes [49]. Intriguingly, adipogenesis impairment appears to proceed with the classical markers of cachexia as well as signs of tissue inflammation [41]. Therefore, factors implicated in the down-regulation of genes involved in pre-adipocyte differentiation may play a key role in the metabolic and functional changes occurring in cachectic adipose tissue.

#### 2.1.3. Extracellular Matrix (ECM) Remodeling

Extracellular matrix remodeling has been demonstrated to play a crucial role in adipogenesis and in the establishment of the tissue structure. Increased fibrosis, observed in adipose tissue of both cancer cachexia murine model [50] and cachectic patients, contributes to the rearrangement of adipose tissue [40]. In adipose tissue, during cancer cachexia, ECM remodeling involves several alterations as changes in collagen deposition, increased amount of infiltrated cells, and insulin resistance. For example, atrophic adipocytes and increased collagen-fibril content were observed in adipose tissue of cachectic MAC16 colon adenocarcinoma-bearing mice [50]. Accordingly, adipose tissue in cachectic patients shows fibrosis due to enhanced synthesis and deposition of collagen fibers [40]. Recently, type I collagen content in subcutaneous adipose tissue of gastrointestinal cancer cachexia patients has been shown to be rearranged, thus leading to increased macrophage and lymphocyte infiltration [37]. In particular, cachectic patients with gastrointestinal cancer display architectural modifications in subcutaneous adipose tissue due to fibrosis and inflammatory cell infiltration surrounding adipocytes in the fibrotic areas [40].

### 2.2. Inflammation and Adipose Tissue Wasting

Cancer-driven chronic inflammation has emerged as a key promotor of cancer cachexia since this pathology is associated with a broad range of metabolic and endocrine impairments, leading to disruptions of tissue function. High circulating levels of pro-inflammatory cytokines, such as tumour necrosis factor α (TNFα) and interleukin 6 (IL-6), have been observed in cachectic patients [51]. Several studies have highlighted that chronic inflammation strongly affects the function of several tissues, including liver, skeletal muscle, and adipose tissue [51]. Adipose tissue inflammation is characterized by the increased recruitment of activated macrophages, particularly evident in visceral adipose tissue depots and in later stages of cachexia [52]. Increased gene expression of activated macrophage markers and inflammatory cytokines, such as IL-6 and TNFα, has been observed in the adipose tissue of cachectic patients with gastrointestinal cancer. Interestingly, increased expression of IL-6 has been positively correlated to the increased circulating levels of IL-6, thus suggesting that adipose tissue, particularly subcutaneous adipose tissue, may act as a key source of inflammatory mediators during cancer cachexia progression [37]. In addition, cachectic patients show fibrosis characterized by the presence of so-called “crown-like structures” (CLS) composed of cluster of differentiation 68 (CD68) positive activated macrophages [53]. The amount of CLS greatly increases in obese mice, in which CLS is surrounded by several WAT macrophages, thus suggesting the formation of “inflammatory” adipocytes in different diseases [54].

In murine models of cancer cachexia, the secretion of pro-inflammatory cytokines by WAT, such as TNFα and IL-6, has been observed. These molecules are able to promote lipid reserve depletion and, consequently, adipose tissue atrophy [37]. Tumour necrosis factor α exerts a key role in the generation of an inflamed state in adipose tissue. Firstly, TNFα suppresses adipocyte differentiation, blocking adipogenic transcription factors, thus strongly impairing adipose tissue cellular turnover [55]. In addition, TNFα inhibits perilipin expression, phospho-proteins located in the surface of lipid droplets, whose function is the blocking of association of lipases to lipids. Hence, perilipin down-regulation leads to lipid depletion and, consequently, the increased circulating levels of free fatty acids, thus inducing insulin resistance in liver and skeletal muscle [6]. Moreover, TNFα inhibits the expression of both GLUT4 and insulin receptor, thus altering glucose transport in adipose cells and decreasing substrates for lipogenesis [56]. Tumour necrosis factor α increases the expression of monocyte chemoattractant protein 1 (MCP1) in adipocytes, resulting in increased infiltrating macrophages and adipose tissue inflammation [6]. Furthermore, TNFα triggers a systemic response, leading to an enhanced circulating level of several cytokines [48], strongly contributing to the onset of chronic inflammation.

For what IL-6 is concerned, circulating IL-6 has been demonstrated to be produced by adipose tissue of cachectic patients [57]. In addition, high levels of IL-6 have been associated with cancer cachexia progression and worsening and correlated with loss of body mass, as well as reduced amounts of adipose tissue [58]. Furthermore, IL-6 has been shown to inhibit the synthesis of lipids in adipocytes [59] and drives the expression of UCP-1, which is important for the WAT to BAT shift occurring in cachexia, responsible for the increased energy expenditure [60].

### 2.3. Adipose Tissue “Browning”

Cancer cachexia has been associated with WAT remodeling characterized by increased immune cell infiltration, altered adipose cell morphogenesis, and progressive conversion of white adipocytes into thermogenic adipocytes (called “browning”), contributing to cachexia progression and high energy expenditure [47,61]. White adipocyte “browning” is characterized by greatly increased levels of brown adipocytes within white adipose depots and has emerged as a key feature of cancer cachexia [61]. The progressive switch from WAT to BAT directly contributes to the increased energy expenditure observed in cachectic patients [42]. Brown adipocytes express a high level of UCP-1, which promotes thermogenesis by uncoupling the mitochondrial electron transport chain from ATP generation. Furthermore, WAT to BAT switch increases lipid mobilization and pro-inflammatory molecules secretion, resulting in a crucial alteration of adipose tissue microenvironment due to increased stromal and inflammatory cell infiltration [42]. Accordingly, cachectic patients exhibit elevated basal energy expenditure levels, increased lipolysis, and adipose tissue wasting [42,61,62].

Host or tumor-derived pro-inflammatory factors strongly contribute to the adipose tissue “browning” and consequently to the onset of this pathology. Among secreted cytokines, IL-6 is one of the main drivers of WAT “browning” [42,61,62,63]. Interleukin-6 inhibits the synthesis of lipids in adipocytes and drives the expression of UCP-1, which is important for the WAT to BAT shift occurring in cachexia, responsible for the increased energy expenditure [59]. The tumor-derived parathyroid hormone-related protein (PTH-rP) is another key promoter of WAT browning. Indeed, PTH-rP promotes the expression of thermogenesis-associated genes in adipose tissue, thus highlighting a crucial role in adipose tissue wasting and increased energy expenditure [42]. In agreement, cachectic patients contain a high level of circulating PTH-rP. Collectively, these data suggest that WAT “browning” mainly occurs in patients affected by PTH-rP high-secreting tumors and that PTH-rP levels positively correlate with increased energy expenditure [42]. Moreover, also ZAG has been demonstrated to be involved in white adipose tissue browning and energy dissipation [64].

## 3. The Involvement of Adipokines in Cancer Cachexia

Cancer cachexia causes inflammation and adipose dysfunction, leading to a dysregulation in synthesis and secretion of several pro-inflammatory and anti-inflammatory adipokines [37,47,65]. However, the role of adipokine dysregulation in cancer-induced cachexia has not yet been fully elucidated. Most studies on this topic have been restricted to adipokine alterations, only correlating their plasma concentrations with tumors, while variations of synthesis, secretion, and signaling in adipose tissue remodeling during cancer cachexia are not in-depth studied so far. The involvement in cancer cachexia of the main molecules secreted by adipocytes has been described in this paragraph. The majority of the published data deal with the potential correlations between circulating leptin, adiponectin, and resistin levels with systemic inflammation and cachexia in obesity-related cancers [66,67,68], while data regarding resistin, visfatin, apelin are more limited.

### 3.1. Leptin

Leptin is a 16 kDa protein discovered in 1994 as a product of the obese gene [69]. Leptin is produced mainly by subcutaneous adipose tissue and, to less extent, by visceral tissue [70]. Serum leptin has a positive correlation with body mass index (BMI) and percent fat ratio, and it is higher in women than in men [71]. This adipokine has a pleiotropic function, ranging from appetite regulation [72] and immune response [73] to angiogenesis [74] and proliferation of different cell types, including cancer cells [75]. Leptin plays a fundamental role in the control of food intake and energy expenditure by regulating appetite at hypothalamic appetite centers [76]. Hyperleptinaemia activates anorexigenic signals, leading to suppression of food intake and stimulation of energy expenditure, whereas low leptin levels act as ‘‘starvation signal’’ through enhanced orexigenic neuropeptide Y (NPY) activity [77]. Furthermore, leptin is involved in many other processes, such as pregnancy, sexual development, bone metabolism, and it is considered an insulin-sensitizing hormone [78]. Biological activities of leptin are mediated through binding to the transmembrane receptor ObR, a member of the class I cytokine receptor superfamily, that includes three classes of isoforms that differ in the length of their intracellular tails: the long OB-Rb, the short OB-Ra, and the soluble sOB-R isoforms. OB-Rb is the major signaling receptor for leptin, located predominantly in the hypothalamus, and also widely distributed in peripherical tissues [79]. Leptin gene expression is up-regulated by pro-inflammatory cytokines, such as TNFα [80] and IL-1 [81], which are also involved in the pathophysiology of cancer cachexia. The correlation between inflammation and leptin level is anyway controversial.

The correlation between leptin levels and cancer differs according to cancer types. Leptin levels are lower in patients with gastric and colon cancers [82,83] whereas higher in patients with breast [84] and ovarian cancers [85]. Since anorexia, hyper-metabolism, and inflammatory acute phase response play a role in the development of cancer cachexia, increased leptin secretion has been suggested to be involved in the onset of this pathology.

Controversial findings have been reported concerning serum leptin levels in cachectic lung cancer patients. Aleman MR et al. studied leptin levels in relation to nutritional status and acute phase response in advanced-stage non-small cell lung cancer [86]. The author reported a lower leptin level in malnourished patients in comparison to control individuals, although the patients had been not divided into cachectic and not cachectic groups. Several published observations are in agreement with a decreased leptin level in lung cancer cachectic patients [65,87,88]. Conversely, some data describe no significant differences in leptin levels in cachectic lung cancer patients in comparison to not cachectic ones [89].

Few studies about gastric cancer have reported a comparison between cachectic and non-cachectic patients regarding leptin serum level. Kerem et al. performed a study including 30 cachectic and 30 non-cachectic patients with gastric cancer, demonstrating that cachectic patients showed increased leptin levels in comparison with control and non-cachectic patients who negatively correlated with BMI [90]. Conversely, no difference in leptin amount was highlighted between cachectic and non-cachectic patients by Huang Q et al. [91]. Moreover, a study considering only cachectic patients has shown an increased level of leptin in patients with respect to healthy subjects [92]. Concerning esophageal cancer, a large study was performed by Diakowska et al. [93]. The authors observed the lowest concentration of leptin in sera from cachectic patients, demonstrating that both leptin and BMI could be considered predictors of cachexia with accuracy exceeding 90%.

Although some studies report that serum leptin is lower in cancer patients in comparison to healthy individuals, few studies have investigated leptin in cancer cachectic conditions [82,94]. Wolf et al. investigated the relationship between cachectic and non-cachectic patients, also considering gender differences [95]. The authors observed that both in men and women, BMI loss was probably associated with leptin level; however, in women, this association was significant and positive, whereas, in men, it was negative and borderline significant. On the other hand, Huang et al. did not find differences between patients [91]. The same results were obtained by Bolukbas et al., who showed no relationship between serum leptin concentration and weight loss percentage [82].

### 3.2. Resistin

Resistin-like molecules (RELMs) are mammalian secreted proteins, identified about 20 years ago, and characterized by several isoforms well described in mouse and human [96]. Among these, human Resistin is a protein of about 12.5 KDa, circulating in the plasma as a dimeric polypeptide (each formed of 92 amino acids) linked by a disulfide bridge. Resistin is mainly produced by peripheral blood mononuclear cells, macrophages, bone marrow, and pancreatic cells [96]. Resistin is also produced by white adipose tissue that contains a low amount of protein [96]. Physiological effects of resistin include potential pro-diabetic and pro-inflammatory activity. In fact, resistin could be considered a linking hormone between obesity and diabetes (“resistin” indicates resistance to insulin) [97]. Resistin effects are mediated by the Toll-like receptor 4(TLR4) receptor, resulting in the activation of the p38 mitogen-activated protein kinase (MAPK), phosphoinositide 3-kinases (PI3K), and nuclear factor-κB (NF-κB) pathways, leading to increased proliferation and angiogenesis. In a recent meta-analysis study, increased plasma resistin level has been linked to an enhanced incidence of obesity-related cancers, such as breast, endometrial, and colorectal cancer, although resistin level cannot be considered a predictor factor [98]. More recently, high serum resistin levels in breast cancer patients have been positively correlated with stage and size of tumor and metastasis [99]. Furthermore, high levels of resistin have been associated also with an increased risk of non-obesity-associated malignancies, such as gastric, colon, and lung cancers [83,88,100,101,102,103]. However, the studies concerning resistin and cancer cachexia are limited at this moment.

The analysis of resistin level in cachectic patients with non-small cell lung cancer (NSCLC) show an increasing tendency of resistin in comparison to non-cachectic ones [88]. In agreement, Karapanagiotou et al. found a significant difference in the resistin level between cachectic and non-cachectic patients [103]. Conversely, Smiechowska et al. reported no differences between cachectic and not cachectic patients [65].

According to two published papers, resistin serum level in patients with gastroesophageal cancer shows an increase in the hormone in cachectic patients [90,93,104]. Moreover, a negative correlation of resistin level with BMI has been reported [93].

### 3.3. Adiponectin

Adiponectin (ApN) is the most abundant adipose-tissue protein with insulin-sensitizing, anti-inflammatory, and antiatherogenic properties [105]. ApN is synthesized mainly in white adipose tissue and secreted in various isoforms with different biological effects on different target tissues. Circulating adiponectin levels (ranging from 2 to 30 µg/mL) primarily depend on genetic, hormonal, inflammatory, dietary, and pharmacological factors. Circulating ApN level negatively correlates with BMI [106,107] and visceral obesity [108]. ApN serum concentration is found to be altered in various diseases. Hypoadiponectinemia has been associated with metabolic syndromes, such as type 2 diabetes, insulin resistance, cardiovascular diseases, and hypertension [109,110]. Besides, hypoadiponectinemia and various genetic polymorphisms have been correlated to an increased risk of obesity-related cancers, such as gastroesophageal, colorectal, prostate, endometrial, and postmenopausal breast cancers [111,112,113,114,115,116]. In recent years, a growing body of in vitro experiments has documented the expression of ApN receptors in several cancer cell lines and has pointed out that the activation of adiponectin signal pathways decreases cancer cell proliferation and promotes apoptosis [112,114,117]. On the other hand, some conflicting studies have provided evidences for a much more complex and contradictory biological role in cancer, with both positive and negative influences on tumor progression [112,114,117,118]. Therefore, further investigations are needed to clarify the pleiotropic functions of ApN in cancer.

The involvement of ApN in the onset of cachexia has been investigated mainly in obesity-related cancers, such as gastrointestinal cancer [37,90,92,104,119,120,121], and in lung cancer [49,65,87,103,118,122,123]. Several papers are clinical studies in which ApN circulating level has been correlated with cancer cachectic conditions.

To date, several clinical studies have reported a decrease in circulating ApN levels in cachectic patients with respect to healthy controls and a positive correlation with BMI loss. However, no clear correlation with clinical-pathological parameters has been detected [92,104]. Unlike these data, Kerem et al. [90] and Batista et al. [37] showed higher serum ApN in cachectic gastric cancer patients with respect to both non-cachectic and healthy controls. In addition, a negative correlation was found between the ApN levels and decreased BMI.

Two clinical studies performed in patients having different types of cancers (colorectal, gastric, pancreas, renal, and prostate cancer) have associated higher serum ApN levels to cachectic patients [37,65]. In particular, Smiechowska et al. determined the adipokines levels and the relation with insulin resistance and inflammatory markers in cachectic cancer patients (colon, lung, prostate, renal cancer) in comparison with non-cachectic cancer patients and healthy controls. ApN levels were higher in both groups of cancer patients with respect to healthy controls, while no differences between cachectic and non-cachectic cancer patients were detected. Moreover, ApN levels did not correlate with insulin resistance, IL-6, and TNF-α levels [65]. Conversely, other studies have pointed out a decrease in serum ApN in cachectic patients with colon cancer and no correlation with BMI loss [92]. Different findings were achieved by Kim et al. in a case-control study, including colorectal and lung cancer patients, both cachectic and non-cachectic [124]. The analysis was carried out at the enrolment and repeated after 2, 4, and 6 months. No significant differences in serum ApN were observed at the enrolment or during the follow-up among the two groups. These data were also confirmed by Wolf et al. in patients with cachectic and non-cachectic breast and colon cancer [95].

To date, several clinical studies have investigated the potential roles of adiponectin in NSCLC cachexia pathogenesis and their relation to systemic inflammatory markers and weight loss, albeit with variable and not exhaustive results [118,123].

In a case-control study of 20 NSCLC patients, Jamieson et al. reported higher circulating ApN levels in cancer cachectic patients with respect to healthy controls. However, no correlation was found between adiponectin and weight loss or markers of systemic inflammation in the cancer group [122]. Similar results were achieved in another clinical study with various types of cancer, including 19 patients with NSCLC [65]. Higher circulating ApN level was detected in cancer patients compared to healthy controls, while no statistically significant differences were found among NSCLC patients with and without cachexia. In addition, ApN levels did not correlate with weight loss or IL-6 and TNF-α levels. In a study of 60 NSCLC patients, ApN concentrations were significantly lower in cancer patients compared to controls [87]. However, no differences were detected among cachectic and non-cachectic patients [87]. Karapanagiotou et al. [103] and Kim et al. [124] pointed out no differences in serum ApN level among NSCLC patients and healthy controls. Besides, the ApN level did not correlate with weight loss. Unlike all these studies, a significant correlation was found between ApN and a nutritional screening questionnaire, namely, the Mini Nutritional Assessment in 115 NSCLC patients [125].

Overall, these studies describe a very complex and unclear role of this adipokine in cancer cachexia. These controversial results may be due to the fact that they have primarily focused on circulating ApN level, which depends on many adipose depots. It is noteworthy that previous studies have demonstrated that different adipose depots undergo to cachexia remodeling in a heterogeneous and time-dependent manner [126,127]. To date, few data correlate adiponectin expression, secretion, and various genetic polymorphisms in different adipose tissue depots with systemic inflammation and weight-loss in cancer cachexia.

#### Adiponectin in Cachectic Adipose Tissue

Regarding adipose tissue in cancer cachexia, Batista et al. confirmed, using animal models for cachexia (rats bearing Walker 256 tumor cells), that there was a marked time-dependent and zonal heterogeneity of response to cachexia among three fat depots (mesenteric, retroperitoneal, and epididymal visceral adipose) [43]. In this study, plasmatic ApN levels were found higher in the early-stage of cachexia, followed by a significant decrease in the late-stage. The increased ApN concentration seemed to be dependent on mesenteric adipose tissue (MEAT). Indeed, in this adipose depot, ApN gene expression was up-regulated during cachexia progression, while in retroperitoneal (RPAT) and epididymal (EAT) depots, ApN gene expression underwent a marked decrease. These gene expression variations were associated also at the protein level. The heterogeneity of MEAT and RPAT response to cachexia was also corroborated by de Oliveira Franco et al. using the same rat models. In particular, in MEAT, they found once again ApN expression enhancement during cachexia progression, while a decrease was observed in RPAT [124].

In a clinical study, Batista et al. investigated some adipokine (adiponectin and leptin) and cytokine (IL-6, TNF-α, IL-10) levels in patients with gastrointestinal cancer cachexia (colorectal, gastric, pancreas cancer) [37]. They correlated the plasma level and changes in mRNA expression of these adipokines in two different adipose tissue depots (subcutaneous, SAT and visceral, VAT) in cachectic and stable weight patients with or without cancer. Regarding adiponectin, the plasma levels were significantly higher in the cachectic groups when compared with stable weight patients. Moreover, adiponectin mRNA expression increased in cancer cachexia patients in SAT, while VAT mRNA expression was unaffected. These findings suggested that subcutaneous adipose tissue is the primary source of plasma ApN changes.

In another clinical study, the remodeling of white adipose tissue in various cancer cachectic patients (colon, gastric, pancreatic cancer) was examined, analyzing the expression profile of lipolysis-related proteins and adipokines [119]. Examined patients included cancer cachexia (CC), weight-stable cancer (WSC) patients, and healthy controls. The circulating ApN level was found to be increased in CC patients in their previous work, as described above [37]. In the present study, ApN gene expression was evaluated in subcutaneous adipose tissue biopsy. The results pointed out a higher expression in CC patient adipose tissue with respect to both controls and WSC patients. To deeply investigate adipose tissue wasting during cancer cachexia progression, plasma ApN levels were analyzed in epididymal white adipose tissue of cachexia rat models (rats bearing Walker 256 tumor cells). The animal models were organized into three groups: control, intermediate cachexia (IC), and terminal cachexia (TC). In contrast to the aforementioned results, in rat models, plasma ApN levels decreased during cachexia progression and particularly in TC. Regarding ApN gene expression, it was higher in IC, while it was decreased in terminal cachexia. This reduction trend was also confirmed at the protein level. In addition, the adiponectin protein concentration was inversely correlated with the TNF-α level in rat epididymal white adipose tissue. The discrepancies observed between patients and animal models have been discussed by assuming that the findings in cachectic cancer patients are consistent with those of animal model intermediate cachexia. Thus, the terminal cachectic animals should be likely compared to refractory cachectic patients. Overall, these findings suggested that, during cancer cachexia, adiponectin undergoes alterations that depend on the stage of the cachexia progression.

In a very recent study, the influence of cancer cachexia on adipogenic genes, including ApN, has been evaluated using a co-culture system [49]. In detail, Lopes et al. co-cultured Lewis lung carcinoma (LLC) tumor cells and an established pre-adipocyte cell line 3T3-L1. The results pointed out that LLC affected the intermediary/late genes related to adipogenesis. Regarding ApN, a decreased secretion in the culture medium after four and eight days of differentiation was observed. Besides, gene expression was down-regulated from eight days. Interestingly, these alterations in ApN gene expression and secretion were associated with an increase in TNF-α secretion, primarily by LLC cells. Finally, the down-regulation of ApN gene expression pointed out in this study corroborated with the results observed in previous works on adipose tissue from animal models of induced cachexia [43,119].

### 3.4. Visfatin

Visfatin is a pleiotropic adipokine mainly secreted by visceral adipose tissue. It was originally discovered as the Pre-B-cell colony enhancing factor (PBEF) by Samal et al. [128] and later as the enzyme catalyzing the rate-limiting step in nicotinamide adenine dinucleotide (NAD) biosynthesis and, therefore, designated as nicotinamide phosphoribosyltransferase (NAMPT) [129]. Visfatin is a pro-inflammatory adipokine with the ability to mimic insulin, and its circulating levels positively correlate with insulin resistance, metabolic syndrome, diabetes, and cardiovascular disease [130]. Besides, a positive correlation has been reported between high visfatin levels with BMI and the size of visceral fat deposits [130]. Emerging evidence has shown a positive correlation between visfatin level and obesity and cancer risk [131,132]. Visfatin has turned out to be overexpressed in various cancers, such as the gastroesophageal, colorectal, prostatic, pancreas, and postmenopausal breast cancer [132,133,134], and has been implicated in cancer proliferation, metastasis, cell angiogenesis, and drug resistance [132]. Nevertheless, other studies have provided conflicting results, showing visfatin levels similar or lower in cancer patients with respect to healthy controls as well as no correlation with cancer risk [121,135].

The information on the role of visfatin in cancer-related cachexia is still limited. To our knowledge, only the study by Silverio et al. reported findings on this issue [119]. The authors analyzed the expression variations of adipokines (leptin, adiponectin, and visfatin) with lipolysis-related proteins in the white adipose tissue of cachectic cancer patients and in rat models of cachexia, as described above. As regards visfatin gene expression, it was significantly higher in cachectic patients and in animal models at the intermediate stage of cachexia but without leading to an up-regulation of both plasma and protein levels. Unlike, in animals, at the terminal stage of cachexia, circulating visfatin, as well as gene and protein expression, was significantly reduced. These alterations of terminal cachexia were in line with those observed for adiponectin and, as hypothesized by the authors, could be linked to the waste of adipose tissue during the progression of cachexia.

### 3.5. Apelin

Apelin is an adipokine identified as a ligand of the G protein-coupled receptor called angiotensin-like-receptor 1 (APJ) [136]. Besides adipose tissue, apelin is also expressed in various tissues, including the central nervous system, gastrointestinal tract, lung, liver, and heart [137], thus suggesting the involvement of the adipokine in several biological processes. Currently, its role as adipokine remains not entirely explained. Emerging findings ascribed to apelin behave similarly to that of insulin as well as pro-inflammatory properties and a role in the regulation of blood pressure [138,139,140,141]. Moreover, it might contribute to obesity-related disorders [138,142] and to the development and progression of cancers [137,143,144]. The expression of apelin has been found to be increased in many types of cancers, where it might act as a potentially proangiogenic factor [137,144,145,146].

To date, not enough information has been published on the role of apelin in cancer-associated cachexia. This issue has been addressed only by Diakowska et al. in a clinical study on gastroesophageal (GEC) cachectic and non-cachectic cancer patients [104]. The aim of the study was to establish the correlation between the level of the adipokines—adiponectin, resistin, apelin—and cancer cachexia. The results pointed out the significantly higher serum and tissue levels of apelin in GEC patients than in healthy controls, mainly in cachectic patients. In addition, apelin was positively associated with serum high sensitivity C-reactive protein (hsCRP) level, suggesting a possible role in the systemic inflammatory response in gastroesophageal cancer. However, no correlation was found between apelin levels and cachexia conditions as well as with any clinical-pathological parameters.

The main adipokine alterations in adipose tissue of cachectic patients are summarized in Table 1.

## 4. Concluding Remarks

Adipose tissue plays a key role in body health due to the secretion of several adipokines, exerting beneficial effects in target tissues. Several pathologies, as cancer cachexia, heavily affect adipokine secretion, and, as a consequence, adipokine circulating level could be highly altered. Hence, adipokine target tissue could have a modified biological response. Actually, few data associate cancer cachexia with adipokines, and, often, published findings are controversial, even within the same cancer type. The understanding of the involvement of adipokines in cancer cachexia could lead to new therapeutic strategies aimed at improving the patient’s quality of life. Approaches designed to regulate adipokine production and the use of adipokine agonist/antagonist could be useful for an initial study aimed at evaluating any beneficial effects on the cachectic adipose tissue. Several studies have demonstrated that these approaches are advantageous for obesity and diabetes care [147,148]. For example, the adiponectin receptor agonist AdipoRon ameliorates insulin resistance and diabetes in mice [149], while the use of apelin antagonist diminishes hepatic fibrosis in rats [150].

Physical exercise could also improve the cachectic condition. Several observations suggest that physical exercise induces beneficial effects in several pathologies like cancer and obesity. Recently, Vulczak et al. showed that in female mice having triple-negative breast cancer, the training induced the smaller tumor mass, higher expression of tumor suppressor genes, and preferential oxidation of carbohydrates in comparison to untrained animals [151]. Moreover, diet combined with aerobic or resistance exercise improves the general functional status in obese older individuals, leading to the preservation of myocellular quality [152,153]. Regarding cancer cachexia, the beneficial effects induced by training exercise is mainly due to its anti-inflammatory role through the decreased levels of circulating pro-inflammatory cytokines [154]. Furthermore, physical exercise affects the amount of the circulating levels of those hormones regulating appetite. For example, training reduces the resistance to the orexigenic hormone ghrelin and increases the circulating levels of the gut hormone peptide tyrosine tyrosine (PYY) [155]. Ultimately, given the importance of adipokines for the well-being of the whole organism, an in-depth study of these molecules in cancer cachexia would be fundamental. This research could lead to the knowledge of adipokine role in the onset of cancer cachexia (in adipose tissue or in adipokine target tissues) or alternatively in the worsening of the pathology.

## Figures and Tables

**Table 1:** The trend of adipokines in different cachexia-inducing tumors are shown. The table indicates cancer type and the decrease/increase of adipokines in cachectic conditions (change in cachexia) and in non-cachectic conditions (change in cancer). The relative references are reported in the brackets.

Adipokine	Cancer Type	Change in Cachexia	Change in Cancer
Leptin	breast	unknown	increase [84]
	colon	unknown	decrease [83]
	gastric	increase [90,92] no change [91]	decrease [82]
	lung	decrease [65,87,88] no change [89]	decrease [89]
	ovarian	unknown	increase [85]
	esophageal	increase [93]	
Resistin	lung	increase [88,103] no change [65]	
Adiponectin	breast	no change [95]	
	colon	increase [37,65] decrease [92] no change [95,124]	
	gastric	increase [37,90]	
	lung	no change [65,87,124] increase [122]	increase [65] decrease [87]
	pancreas	increase [37,65]	
	prostate	increase [37,65]	
	kidney	increase [37,65]	
Visfatin	breast		increase [131,133]
	colorectal	increase [119]	increase [131,133]
	gastroesophageal	increase [119]	increase [131,133]
	pancreas	increase [119]	increase [131,133]
	prostate		increase [131,133]
Apelin	gastroesophageal	increase [104]	increase [104]
